# Editorial: Diagnosis of neuromuscular disorders in the era of personalized genomic medicine

**DOI:** 10.3389/fneur.2023.1188037

**Published:** 2023-05-24

**Authors:** Mohamed Kazamel, Cheng-Ying Ho

**Affiliations:** ^1^Department of Neurology, University of Alabama at Birmingham, Birmingham, AL, United States; ^2^Department of Pathology, Johns Hopkins University School of Medicine, Baltimore, MD, United States

**Keywords:** acute hepatic porphyria, amyloidosis, inclusion body myositis, precision medicine, whole exome sequencing

In the last decade, the evolution of diagnostic technologies, such as next-generation sequencing (NGS), has drastically improved the diagnostic yield and changed how physicians approach and manage neuromuscular disorders. However, employing these modern techniques to diagnose and treat neuromuscular disorders is not without its challenges, e.g., the cost-benefit issue, difficulties in handling and interpreting the genetic data, and the gap between knowledge and action; they have all limited the utility of these novel tools in the care of patients with neuromuscular conditions. The articles in this Research Topic aimed to develop a systemic approach to guide physicians in efficiently utilizing personalized genomic medicine in the management of specific neuromuscular disorders, e.g., hereditary and idiopathic myopathies such as inclusion body myositis (IBM) and inherited neuropathies.

Ng et al. discussed the benefits and limitations of using genetic panels as first-tier investigations in the evaluation of neuromuscular disorders. This approach could shorten diagnostic delays and end the need for invasive tests, such as muscle or nerve biopsies (1). Early molecular diagnosis leads to a more specific treatment, which is essential in certain disorders, such as congenital myasthenic syndromes and Duchenne muscular dystrophy (DMD), in which certain medications can be harmful or ineffective (2). Compared with whole-genome sequencing (WGS), genetic panels are less likely to generate incidental findings that are not pertinent to the clinical presentation (3). Additionally, they have the capacity to evaluate large genes that cannot be reliably tested by WGS, e.g., *TTN, NEB*, and *RYR1* (4). However, NGS-based gene panels are incapable of evaluating certain elements of the human genome, such as repeats (5), and have variable sensitivity in detecting deep intronic variants and copy number variations, as in some cases of DMD and *PMP22*-related Charcot–Marie–Tooth (CMT) disease, respectively (6, 7). Additionally, the authors argued against using genetic panels as first-tier tests in patients with dual pathology, e.g., neuromyopathies or diseases with poor genotype-phenotype correlation. Finally, the authors offered recommendations on how to efficiently and accurately utilize the genetic panels in the disease work-up to maximize the benefits.

A mini-review by Kazamel et al. reviewed the clinical presentation, pathophysiology, and management of acute hepatic porphyrias (AHPs). AHPs are a group of inherited disorders characterized by hepatic overproduction of the neurotoxic porphyrin precursors δ-aminolevulinic acid (ALA) and porphobilinogen (PBG) (8). Patients report severe abdominal pain during attacks (9) and recent studies reported neuropathic pain in many patients in between attacks (10). The authors recommended screening of any patient with recurrent severe acute abdominal pain with no obvious explanation for AHPs, especially if it is associated with neuropathic pain, autonomic manifestations, or encephalopathy. Checking spot urine PBG adjusted to creatinine during the attack can inform the diagnosis, which nevertheless needs to be confirmed by genetic testing (11). Autonomic neuropathy is likely responsible for most acute symptoms. The blood-nerve-barrier (BNB) is absent/less restrictive in the autonomic ganglia and gut small nerve fibers, rendering these sites more vulnerable to ALA neurotoxic effects (8). The observation that lower back pain often precedes limb pain suggests that nerve roots, where the BNB is also less restrictive, are a primary site of pathology. Potential mechanisms of chronic pain include chronic small fiber neuropathy due to ALA neurotoxicity in addition to peripheral and central sensitization elicited by hepatic proinflammatory mediators (12, 13). While pain during attacks is treated with opiates and hemin infusions, the authors recommended treating pain between attacks with gabapentinoids and certain antidepressants before opiates. A recently approved siRNA molecule, givosiran, reduces ALA and PBG levels and likely helps with chronic pain (14). Early diagnosis with biochemical and genetic testing and specific treatment are essential for preventing long-term neurologic morbidity.

An article by Naddaf provided a comprehensive review of IBM, the most common form of inflammatory myopathy in patients over the age of 40 years. The review discussed the unique characteristics of the disease, including clinical, radiological, electrodiagnostic, and pathological features. It also discussed the long-term outcomes as well as the challenges in establishing the diagnosis and treating the disease. Despite the fact that no pharmacological treatments are currently available for IBM, in the era of personalized medicine, individualized outcome measures and clinical trial designs offer an innovative approach for addressing this issue. The disease mechanism is not well-understood, possibly due to the lack of an appropriate animal model (15). However, based on human data, inflammation and abnormal protein aggregates, e.g., TDP-43, may both play a role in the pathogenesis of the disease (16, 17).

A review by Bumma et al. discussed a single tertiary center's experience with the multidisciplinary management of patients with amyloidosis in the era of precision medicine. Additionally, the authors briefly reviewed the pathophysiology and management of different types of amyloidosis. They recognized the lack of coordinated care for these patients, which has led to a delay in diagnosis and treatment, with 75% of cases taking 12 months to reach a definitive diagnosis (18). In their comprehensive amyloid clinic, different specialties work collaboratively to manage patients' symptoms, sustain the involved organs, and reduce the amyloid burden. A board-certified hematologist directs the clinic and works with specialists from other medical disciplines, including nephrology, neurology, cardiology, and physical therapy. The clinic workflow follows a one-stop design, in which patients are seen in the same room by rotating providers. The program's ultimate goal is to improve access and outcome for amyloidosis patients through the delivery of personalized medicine.

Lastly, Jiang et al. reported axonal motor-predominant neuropathy and myopathy with rimmed vacuoles in a young male and his brother. The patient and his brother both harbored a novel and likely pathogenic homozygous mutation p.I63N (c.188T>A) in the gene histidine triad nucleotide-binding protein 1 (*HINT1*). Recessive mutations in HINT1 have been associated with axonal motor-predominant CMT disease with neuromyotonia (19). The presence of rimmed vacuoles on the muscle biopsy raises the possibility that mutations in the *HINT1* gene may also cause myopathy.

In summary, the articles in this Research Topic provide successful personalized medicine examples of certain neuromuscular disorders. The Research Topic described the use of genetic panels as first-tier tests for inherited neuromuscular diseases, which led to unnecessary tests and ineffective treatments for certain muscular dystrophies being avoided. Additionally, the Research Topic featured the recent FDA approval of specifically designed siRNA molecules for treating inherited neuropathies like those associated with AHPs and hereditary TTR amyloidosis, with a particular focus on the multidisciplinary mode of management for the latter. It indeed emphasized the need for individualized outcome measures and innovative clinical trial designs for potential IBM therapeutics. Ultimately, we hope that a better understanding of personalized genomic medicine will increase the use of molecular diagnostic tools, improve the diagnostic yield, and maximize the treatment benefit for more neuromuscular diseases.

## Author contributions

MK and C-YH writing and editing the original draft. Both authors contributed to the article and approved the submitted version.
